# Supplementary material for: Thionated levofloxacin derivative: Potential repurposing for cancer treatment and synergism with doxorubicin on doxorubicin-resistant lung cancer cells
Source: PLoS One. 2025 Jun 9;20(6):e0324930. doi: 10.1371/journal.pone.0324930 (PMC12148111; doi:10.1371/journal.pone.0324930)
Supplement: Attachment — Submitted filename: PONE-D-25-14330_with side bubble comments.doc [file pone.0324930.s001.doc]

PLOSONE

**Thionated Levofloxacin Derivatives: Potential Repurposing for Cancer Treatment andSynergismwithDoxorubicinonDoxorubicin-ResistantLungCancerCells**

--ManuscriptDraft--

| **ManuscriptNumber:** | PONE-D-25-14330 |
| --- | --- |
| **ArticleType:** | ResearchArticle |
| **FullTitle:** | ThionatedLevofloxacinDerivatives:PotentialRepurposingforCancerTreatmentand Synergism with Doxorubicin on Doxorubicin-Resistant Lung Cancer Cells |
| **ShortTitle:** | RepurposingThionatedLevofloxacinDerivativesforCancerTreatment |
| **CorrespondingAuthor:** | OsamaAbusara  Al-ZaytoonahUniversityofJordanFacultyofPharmacy Amman, JORDAN |
| **Keywords:** | cancer;DOX;repurposing;synergism;thionatedlevofloxacin |
| **Abstract:** | Objectives: Fluoroquinolones, such as levofloxacin (LVX), are extended-spectrum drugsusedforthetreatmentofbacterialinfections.Severalfluoroquinolonederivatives have shown promising antibacterial and anticancer activities. Our group has earlier synthesized and investigated thionated LVX analogs, compounds 2 and 3, on A549 (non-small cell lung cancer) cell line and showed promising anticancer activity. In this study, compounds 2 and 3 were evaluated on prostate (PC-3), breast (MCF7), colorectal (Caco-2), and small cell lung cancer (H69 and H69AR) cell lines. Methods: The anticancer activity was measured using resazurin colorimetric method.  Combination treatments with doxorubicin (DOX) were also employed and combination index (CI) values were calculated. Results: Compound 3 possessed higher anticancer activity compared to compound 2 on the tested cancer cell lines with the highest activity on PC-3 cells with IC50 value of 3.58 µM. The mechanism of cytotoxicity may be, in part, via aldehyde dehydrogenase enzyme inhibition. Combination treatment with DOX resulted in significant reduction of cell viability in PC-3, H69, and H69AR (DOX- resistantformofH69)cells,withthoseonH69andH69ARcellsresultedinadditive(CI  = 1.0) and synergistic effects (CI = 0.6), respectively. Conclusions: Compound 3, a thionated LVX derivative, showed a promising anticancer activity, prompting its potentialrepurposingforcancertreatmentaswellascombinationtreatmentwithDOX on DOX-resistant cancer cells. |
| **OrderofAuthors:** | HamzaAbumansour |
|  | OsamaH.Abusara |
|  | MohammadAbu-Sini |
|  | WiamKhalil |
|  | AliI.M. Ibrahim |
|  | AmalM.Badawoud |
|  | MajedS.AlYami |
|  | DinaH.Abulebdah |
|  | ShirazHalloush |
| **AdditionalInformation:** |  |
| **Question** | **Response** |
| **FinancialDisclosure**  Enterafinancialdisclosurestatementthat describes the sources of funding for the work included in this submission. Review the submission guidelinesfor detailed | Yes |

| requirements.Viewpublishedresearch articles from *PLOS ONE*for specific examples.  Thisstatementisrequiredforsubmission and**willappearinthepublishedarticle**if thesubmissionisaccepted.Pleasemake sure it is accurate. |  |
| --- | --- |
| **Fundedstudies**  Enterastatementwiththefollowingdetails:  Initialsoftheauthorswhoreceivedeach award  Grantnumbersawardedtoeachauthor  Thefullnameofeachfunder  URLofeachfunder website  Didthesponsorsorfundersplayanyrolein the study design, data collection and analysis,decisiontopublish,orpreparation of the manuscript? |  |
| Didyoureceivefundingforthiswork? |  |
| Pleaseaddfundingdetails.  asfollow-upto"**FinancialDisclosure**  Enterafinancialdisclosurestatementthat describes the sources of funding for the work included in this submission. Review the submission guidelinesfor detailed requirements. View published research articles from *PLOS ONE*for specific examples.  Thisstatementisrequiredforsubmission and**willappearinthepublishedarticle**if thesubmissionisaccepted.Pleasemake sure it is accurate. | This work was supported by Al-Zaytoonah University of Jordan grants (30/06/2024- 2025 and 27/06/2024-2025). Princess Nourah bint Abdulrahman University ResearchersSupportingProjectnumber(PNURSP2025R418),PrincessNourahbint Abdulrahman University, Riyadh, Saudi Arabia. |

| **Fundedstudies**  Enterastatementwiththefollowingdetails:  Initialsoftheauthorswhoreceivedeach award  Grantnumbersawardedtoeachauthor  Thefullnameofeachfunder  URLofeachfunder website  Didthesponsorsorfundersplayanyrolein the study design, data collection and analysis,decisiontopublish,orpreparation of the manuscript? |  |
| --- | --- |
| Didyoureceivefundingforthiswork?" |  |
| Please select the country of your main researchfunder(pleaseselectcarefully as in some cases this is used in fee calculation).  asfollow-upto"**FinancialDisclosure**  Enterafinancialdisclosurestatementthat describes the sources of funding for the work included in this submission. Review the submission guidelinesfor detailed requirements. View published research articles from *PLOS ONE*for specific examples.  Thisstatementisrequiredforsubmission and**willappearinthepublishedarticle**if thesubmissionisaccepted.Pleasemake sure it is accurate. | JORDAN-JO |
| **Fundedstudies**  Enterastatementwiththefollowingdetails:  Initialsoftheauthorswhoreceivedeach award  Grantnumbersawardedtoeachauthor  Thefullnameofeachfunder  URLofeachfunder website  Didthesponsorsorfundersplayanyrolein the study design, data collection and analysis,decisiontopublish,orpreparation of the manuscript? |  |
| Didyoureceivefundingforthiswork?" |  |
| **CompetingInterests** | Theauthorsdeclarethattheyhavenoconflictofinteresttodeclare. |

| Use the instructions below to enter a competing interest statement for this submission. On behalf of all authors, disclose any competing intereststhat couldbeperceivedtobiasthiswork—acknowledgingallfinancialsupport and any other relevant financial or non- financial competing interests.  This statement is required for submission and**willappearinthepublishedarticle**if the submission is accepted. Please make sure it is accurate and that any funding sourceslistedinyourFundingInformation later in the submission form are also declared in your Financial Disclosure statement.  Viewpublishedresearcharticlesfrom  *PLOSONE*forspecific examples. |  |
| --- | --- |
| **NOauthorshavecompetinginterests**  Enter:*Theauthorshavedeclaredthatno competing interests exist.*  **Authorswithcompeting interests**  Entercompetinginterestdetailsbeginning with this statement:  *I have read the journal's policy and the authorsofthismanuscripthavethefollowing competing interests: [insert competing interests here]* |  |
| *typeset |  |
| **EthicsStatement**  Enter an ethics statement for this submission.Thisstatementisrequiredif the study involved:  Humanparticipants  Humanspecimensor tissue  Vertebrateanimalsorcephalopods  Vertebrateembryosortissues | N/A |

| Fieldresearch  Write"N/A"ifthesubmissiondoesnot require an ethics statement.  General guidance is provided below. Consult the submission guidelinesfor detailed instructions. **Make sure that all information entered here is included in the Methodssectionofthemanuscript.** |  |
| --- | --- |

| **Formatforspecificstudytypes**  **HumanSubjectResearch(involvinghuman participantsand/ortissue)**  Give the name of the institutional review boardorethicscommitteethatapprovedthe study  Includetheapprovalnumberand/ora statement indicating approval of this research  Indicate the form of consent obtained (written/oral)orthereasonthatconsentwas not obtained (e.g. the data were analyzed anonymously)  **Animal Research (involving vertebrate animals,embryosortissues)**  ProvidethenameoftheInstitutionalAnimal CareandUseCommittee(IACUC)orother relevant ethics board that reviewed the study protocol, and indicate whether they approved this research or granted a formal waiver of ethical approval  Includeanapprovalnumberifonewas obtained  If the study involved *non-human primates*, add*additionaldetails*aboutanimalwelfare and steps taken to ameliorate suffering  If anesthesia, euthanasia, or any kind of animalsacrificeispartofthestudy,include briefly which substances and/or methods were applied  **FieldResearch**  Include the following details if this study involvesthecollectionofplant,animal,or other materials from a natural setting:  Fieldpermitnumber  Nameoftheinstitutionorrelevantbodythat granted permission |  |
| --- | --- |
|  |  |
| **DataAvailability**  Authors are required to make all data underlying the findings described fully available,withoutrestriction,andfromthe time of publication. PLOS allows rare exceptions to address legal and ethical concerns.SeethePLOSDataPolicyand FAQfor detailed information. | Yes-alldataarefullyavailablewithoutrestriction |

| A Data Availability Statement describing wherethedatacanbefoundisrequiredat submission.Youranswerstothisquestion constitute the Data Availability Statement and**willbepublishedinthearticle**,if accepted. |  |
| --- | --- |
| **Important:** Stating ‘data available on request from the author’ is not sufficient. If your data areonlyavailableuponrequest,select‘No’for thefirstquestionandexplainyourexceptional situation in the text box. |  |
| Do the authors confirm that all data underlyingthefindingsdescribedintheir manuscript are fully available without restriction? |  |
| **Describewherethedatamaybefoundin fullsentences.Ifyouarecopyingour sampletext, replaceany instancesofXXX withtheappropriatedetails.**  Ifthedataare**heldorwillbeheldina publicrepository**,includeURLs, accession numbers or DOIs. If this information will only be available after acceptance,indicatethisbytickingthe box below. For example: *All XXX files are available from the XXX database (accession number(s) XXX, XXX.*).  Ifthedataareallcontained**withinthe manuscriptand/orSupporting Informationfiles**,enterthefollowing: *All relevant data are within the manuscript and its Supporting Information files.*  Ifneitheroftheseappliesbutyouare able to provide **details of access elsewhere**,withorwithoutlimitations, please do so. For example:  *Datacannotbesharedpubliclybecause of [XXX]. Data are available from the XXX Institutional Data Access / Ethics Committee (contact via XXX) for researchers who meet the criteria for access to confidential data.*  *The data underlying the results presented in the study are available from(includethenameofthethirdparty* | Thedatasetsgeneratedduringand/oranalyzedduringthecurrentstudyarepresented within the manuscript. |

| *andcontactinformationorURL).*  This text is appropriate if the data are ownedbyathirdpartyandauthorsdo nothavepermissiontosharethedata.  *typeset |  |
| --- | --- |
| Additionaldataavailabilityinformation: |  |

Manuscript	Clickheretoaccess/download;Manuscript;Manuscript_after technical checks.docx

ThionatedLevofloxacinDerivatives:PotentialRepurposingforCancerTreatmentand

**SynergismwithDoxorubicinonDoxorubicin-ResistantLungCancerCells**

3

HamzaAbumansour^a,§,*^,OsamaH.Abusara^a,§^,MohammadAbu-Sini^a^,WiamKhalil^b^,AliI.M.

Ibrahim^a^,AmalM.Badawoud^c^,MajedS.AlYami^d,e,f^,DinaH.Abulebdah^a^,ShirazHalloush^g^

6

^a^DepartmentofPharmacy,FacultyofPharmacy,Al-Zaytoonah UniversityofJordan, P.O.Box130,

Amman11733,Jordan.

^b^DepartmentofPharmacology,SchoolofMedicine,TheUniversityofJordan,Amman11942,

Jordan.

^c^DepartmentofPharmacyPractice,CollegeofPharmacy,PrincessNourahbintAbdulrahman

University,P.O.Box84428,Riyadh11671,SaudiArabia

^d^DepartmentofPharmacyPractice,CollegeofPharmacy,KingSaudbinAbdulazizUniversityfor

HealthSciences,P.O.Box3660,Riyadh11481,SaudiArabia

^e^KingAbdulazizMedicalCity,NationalGuardHealthAffairs,Riyadh,SaudiArabia

^f^KingAbdullahInternationalMedicalResearchCenter,Riyadh,SaudiArabia

^g^DepartmentofClinicalPharmacyandTherapeutics,FacultyofPharmacy,AppliedSciencePrivate

University,P.O.Box541350,Amman11937,Jordan

^§^Theseauthorscontributed equallyto thiswork.

^*^CorrespondingAuthor:h.abumansour@zuj.edu.jo(HamzaAbumansour).

21

22	SubmittingAuthor:o.abusara@zuj.edu.jo(Osama H.Abusara)

23

Authors’Emailsand ORCiDs:

•	HamzaAbumansour:(h.abumansour@zuj.edu.jo)(https://orcid.org/0000-0003-3015-9859)

•	OsamaH.Abusara:(o.abusara@zuj.edu.jo)(https://orcid.org/0000-0002-0856-5618)

•	Mohammad Abu-Sini:(mohammad.abusini@zuj.edu.jo)(https://orcid.org/0000-0001-7352-

28	9739)

•	WiamKhalil:(w.khalil@ju.edu.jo)

•	AliI.M.Ibrahim:(a.ibrahim@zuj.edu.jo)(https://orcid.org/0000-0002-8668-4647)

•	Amal	M.	Badawoud:(AMBadawoud@pnu.edu.sa)(https://orcid.org/0000-0001-7718-

32	562X)

•	MajedS. AlYami:(Yamim@ksau-hs.edu.sa)(https://orcid.org/0000-0003-2308-8407)

•	DinaH.Abulebdah: (denaabulebdah@gmail.com)

•	ShirazHalloush:(s_halloush@asu.edu.jo)(https://orcid.org/0000-0003-3966-2733)

36

37

38

39

40

41

42

43

44

45

46

47

48

49

50

51

52

Abstract

**Objectives:**Fluoroquinolones,suchaslevofloxacin(LVX),areextended-spectrumdrugs

usedforthetreatmentofbacterialinfections.Severalfluoroquinolonederivativeshaveshown

promisingantibacterialandanticanceractivities.Ourgrouphasearliersynthesizedandinvestigated

thionatedLVXanalogs,compounds**2**and**3**,onA549(non-smallcelllungcancer)celllineand

showedpromisinganticanceractivity.Inthisstudy,compounds**2**and**3**wereevaluatedonprostate

(PC-3),breast(MCF7),colorectal(Caco-2),andsmallcell lungcancer (H69andH69AR)cell lines.

**Methods:**Theanticanceractivitywasmeasuredusingresazurincolorimetricmethod.Combination

treatmentswithdoxorubicin(DOX)werealsoemployedandcombinationindex(CI)valueswere

calculated.**Results:**Compound**3**possessedhigheranticanceractivitycomparedtocompound**2**on

thetestedcancercelllineswiththehighestactivityonPC-3cellswithIC50valueof3.58µM.The

mechanismofcytotoxicitymaybe,inpart,viaaldehydedehydrogenaseenzymeinhibition.

CombinationtreatmentwithDOXresultedinsignificantreductionofcellviabilityinPC-3,H69,and

H69AR(DOX-resistantformofH69)cells,withthoseonH69andH69ARcellsresultedin additive

(CI=1.0)andsynergisticeffects(CI=0.6),respectively.**Conclusions:**Compound**3**,athionated

LVXderivative,showedapromisinganticanceractivity,promptingitspotentialrepurposingfor

cancertreatmentaswell ascombinationtreatment withDOXonDOX-resistantcancercells.

70

71	**Keywords:**cancer,DOX,repurposing,synergism,thionatedlevofloxacin

72

73

74

75

76

77

78

1. Introduction

Fluoroquinolonesareaclassofbroad-spectrumantibioticscommonlyusedtotreatavarietyof

bacterialinfections.TheyexerttheirantibacterialactivitybyinhibitingbacterialDNAgyrase,

topoisomeraseII,andtopoisomeraseIVenzymes,whichareessentialforbacterialnucleicacids

functionsandrepairprocesses,andthusforproliferation[1,2].Amongothers,thecommonlyused

fluoroquinolonesclinicallyarelevofloxacin(LVX),ciprofloxacin,andmoxifloxacin[2].Theyare

extended-spectrumantibioticclassthatactagainstinfectionscausedbygram-positiveandgram-

negativebacteria,includingurinarytractinfections[3],respiratorytractinfection[4],boneinfections

[5],sexuallytransmitteddiseases [6],and skininfections[7].

Fluoroquinoloneshavealsobeeninvestigatedforotherbiologicalactivitiesapartfrom

antibacterialeffects.Forexample,Huang*etal*.showedthatagroupoffluoroquinolonesincluding

ciprofloxacin, LVX,clinafloxacin,gatifloxacin,andenrofloxacin,effectivelysuppressedTGF-βand

PMA-induced MMP-9 levelsandactivityinHepG2andA549cancercellculturesinaconcentration

andtime-dependentmanner[8].Furthermore,thesefluoroquinolonesinhibitedTGF-βandPMA-

inducedcellmigrationbytargetingthep38and cyclicAMPsignalingpathways [8].

Newerfluoroquinolonesderivativeshavebeensynthesizedandinvestigatedasapotentialwayto

improvetheirefficacy,theirantimicrobialspectrum,andforcancertreatmentrepurposingeffortsvia

introducingseveralmodificationsonthequinolonenucleus.Forexample,Khan*etal*.,showedthat

chloroquine,anexampleofdrugwithaquinolonenucleus,entrappedinphosphatidylserineliposomes

haveincreasedactivityagainst*Cryptococcusneoformans*fungalinfectionsbothin*invitro*and*invivo*

studiesasasingletreatmentorincombination withfluconazole (FLZ),aknownantifungaldrug[9].

OtherexamplesarethionatedLVXderivatives,suchascompounds**2**and**3**(**Figure1**)synthesized

byourgroup,thathaveshownpromisingactivityagainstbacteriaaswellascancercells,*invitro*[10,

11].

103

104

| 105 | **(Figure1 here)** |
| --- | --- |
| 106 |  |
| 107 | Otherderivativeshavebeeninvestigatedfortheirmechanismofcytotoxicityandantiproliferative |
| 108 | effects.Theseincludearrestingthecellcycleandinterferingwithitsphases,inducingcelldeath, |
| 109 | suppressingangiogenesis,interferingwithcellmigration,andadjustingcellsignalingpathways [12]. |
| 110 | Thesemodifiedquinolonederivativesthathaveshowncytotoxicandantiproliferativeeffectson |
| 111 | variouscancercelllinesinclude:quinolonederivativescontainingmorpholinalkylaminosidechains |
| 112 | [13],5-chloroquinolin-8-olderivatives[14],andLVXcarboxamidesderivativesinwhich3-chloroor |
| 113 | 4-fluorosubstituentontheS-benzylmoietyhadpositiveantiproliferativeeffectcomparedto |
| 114 | doxorubicin[15]. |
| 115 | Recently,apreviousstudybyourgroup[11]examinedthecytotoxicityandthepotential |
| 116 | mechanismofathionatedLVXderivative(compound**3**)onA549cellline.Itshowedapromising |
| 117 | anticanceractivitywithasuggestedmechanismviaaldehydedehydrogenase(ALDH)enzyme |
| 118 | inhibition[11].Additionally,theanticancereffectofcompound**3**onA549celllinehasbeen |
| 119 | investigatedwhenithasbeencombinedwithdoxorubicin(DOX)andshowedenhancedactivity[11]. |
| 120 | Inthisstudy,weaimedtofurtherinvestigatetheanticancerchemotherapeuticspectraof |
| 121 | compounds**2**and**3**.Herein,severalcancercelllineswereused;prostatecancercellline(PC-3), |
| 122 | breastcancercellline(MCF7),colorectalcancercellline(Caco-2),andsmallcelllungcancer |
| 123 | (SCLC)celllines(H69anditsDOX-resistantformH69AR).Asfortheanticanceractivity,wealso |
| 124 | investigatedthecombinationofDOXandthepossibilityofsynergism,knowingthatseveralstudies |
| 125 | investigatecombinationtreatmentsasawaytoinducesynergism[11,16-21]. |
| 126 | **2.MaterialsandMethods** |
| 127 | **2.1.CellCulture** |
| 128 | DMEM–highglucose,andRPMI-1640MediumwereobtainedfromEuroclone,Italy.Heat |
| 129 | inactivatedFetalBovineSerum(FBS)wasobtainedfromCapricornScientific,Germany.Prostate |
| 130 | cancercellline(PC-3(ATCCCRL-1435)),breastcancercellline(MCF7(ATCCHTB-22)),colon |
| 131 | cancercellline(Caco-2[Caco2](ATCCHTB-37)),andSCLCcelllines(H69(NCI-H69[H69] |

(ATCCHTB-119))andH69AR(ATCCCRL-11351))wereobtainedfromATCC.Completemedium

wasusedforcellgrowth;PC-3,MCF7,andH69cellsin10%(v/v)FBS/RPMI,H69ARcells20%

(v/v)FBS/RPMI,andCaco-2cellsin10%(v/v)FBS/DMEM.Cellswereincubatedat37°C.With

theexceptionofthesuspendedH69 cells,allothercellsareadherent,inwhichmedia wasaspirated,

cellswashedwithPBS,andfreshcompletemediumwasaddedfortheirgrowth.H69cellswere

continuouslyresuspended in fresh medium.

2.2.Cellviabilityassaysusingresazurindyemethod

ResazurinsodiumsaltandDOXwereobtainedfromSigma-Aldrich,USA.Ourgrouphas

synthesizedcompounds**2**and**3**[10].Theresazurindyecolorimetricmethodwasusedtocarryout

thecellviabilityassaysasdescribedbefore[11,22].Briefly,theseedingdensityforPC-3,MCF7,

Caco-2,andH69ARcellsin96-wellplateswas1×10^4^cells/200μL/well,whereasH69cells’seeding

densitywas1×10^4^cells/180μL/well.Fortheadherentcells,24hpost-seeding,aspirationoftheold

mediumandtheadditionof180µLoffreshmediumwereperformed.Thetestedcompounds(**2**,**3**,

andDOX)wereinitiallydissolvedinDMSOanddilutedwithcompletemedium(ofeachcellline)

formingseveralstocksolutionswithconcentrationsbeingrangedfrom1mMto0.03µM.Then,20

μLofthecompounds’stocksolutionswasaddedandtreatmentswerefor96h.Wellsservingas

untreatedcontrolandforbackgroundfluorescencewereprepared.Following96hincubation,

resazurindye(20μL;0.125mg/mLinPBS)wasaddedontowellsandplatesincubatedfor4h.

BioTeKSYNERGYHTXplatereaderwasthenusedtorecordthefluorescencereadingsat540nm

(excitation)and620nm(emission)forpercentage viabilitycalculationscomparedtothecontrol.

2.3.UsingDoxorubicinfortheCombinationTreatments

Cellviabilityassaysfor thecombination treatmentswereperformedasmentionedaboveand

aspreviouslydescribed[11,19].ThefinalwellconcentrationofDOXusedwasatoraroundthe IC50

valueforeachcellline.Thefinalwellconcentrationsusedforcompound**3**rangedfrom12.5to0.391

µM.

2.4.CombinationIndicesfortheCombinationTreatments

Cellviabilityassayswereperformedasmentionedaboveandaspreviouslydescribed [11].A

stocksolution(10X)forcompound**3**aswellasforDOXwerepreparedattheirrespectiveIC50

concentrationusingcompletemedium.The10Xstocksolutionofcompound**3**wasmixedwiththe

DOX’s10Xstocksolutionata1:1ratioformingtheinitialworkingconcentration.Theinitialworking

concentrationwasdiluted7timesat1:1ratiousingcompletemedium.Fortheadherentcells,medium

wasaspiratedand200µLofeachmixturewasaddedandplatesincubatedfor96h.Forthesuspended

cells(H69),thesameprocedurewasappliedbutwithinitiallypreparing100Xstocksolutionand20

µLofthedilutedmixtureswereaddedto180µLofseededcells.Later,resazurinassaywasconducted

166

167

asmentionedabove.The followingequationwasusedtocalculatethecombinationindices [11, 20]:

𝐷_1_	𝐷_2_

𝐶𝑜𝑚𝑏𝑖𝑛𝑎𝑡𝑖𝑜𝑛𝐼𝑛𝑑𝑒𝑥(𝐶𝐼)=	+

𝐷𝑥1	𝐷𝑥2

D1andD2arethenewIC50valuesforthecompoundsinthecombinationtreatment,whileDx1

andDx2aretheoriginalIC50valuesforthesamecompoundswhenusedalone.

2.5.StatisticalAnalysis

Dataareshownasmeansandstandarderrorsofthemeans(SEM).TheIC50valueswere

calculatedusingnonlinearregressionanalysis.Theanalysisofthecombinationexperiments

(compound+DOX)withDOXalonewasperformedusingone-wayANOVAfollowedbyTukey’s

multiplecomparisonsanalysis.GraphPadPrismversion9.0wasusedtocarryoutthe analysis.

3.Resultsand Discussion

**3.1.CellViability Assays**

ExperimentswereconductedbytreatingPC-3,MCF7,Caco-2,H69,andH69ARcelllines

for96hwithDOXandcompounds**2**and**3**.DOXwasusedforitsknownanticancerefficacyagainst

severaltypesofcancers[23-25]andforcomparisonpurposes.Compounds**2**and**3**werepreviously

investigatedfortheiranticanceractivityonothercancercelllines[10,11]andwerefurther

investigatedinthisstudyonapanel ofcancercelllines.Theresultsarepresentedin**Table1**.

182

**Table1.**Cytotoxicity(IC50±SEM)ofDOXandcompounds**2** and**3**onPC-3,MCF7, Caco-2, H69,

andH69ARcelllinesaftertreatmentfor96h;n= 3.

| **Compounds** | **PC-3 IC50 ±SEM**  **(μM)** | **MCF7 IC50±SEM**  **(μM)** | **Caco-2**  **IC50 ±SEM (μM)** | **H69 IC50±SEM**  **(μM)** | **H69AR**  **IC50±SEM (μM)** |
| --- | --- | --- | --- | --- | --- |
| **DOX** | 0.35 ± 0.02 | 0.45 ± 0.03 | 0.28 ± 0.03 | 0.09 ± 0.01 | 4.62 ± 0.79 |
| **Compound2** | 21.42 ± 1.26 | >100 | >100 | >100 | >100 |
| **Compound3** | 3.58 ± 0.16 | >100 | 52.34 ± 3.97 | 14.20 ± 0.28 | 35.50 ± 0.54 |

| 185 | SEM:standarderrorofthemean; DOX:doxorubicin |
| --- | --- |
| 186 |  |
| 187 | **Table1**showsthatDOXhasthehighestcytotoxiceffectonalltestedcancercelllines, |
| 188 | comparedtoothercompounds,duetoitseffectiveanticanceractivityagainstseveralcancers,such |
| 189 | prostate,breast,andcolorectalcancers[23-25].Ourdata(**Table1**)showsthatDOX’shighestactivity, |
| 190 | amongtestedcancer celllines,wasrecordedagainsttheSCLCcelllineH69withIC50valueof0.09 |
| 191 | µM,whichis~50timesmoresensitivecomparedtoitsresistantform;H69AR[26-28],withIC50 |
| 192 | valueof4.62 µM |
| 193 | TherewereseveralLVXderivativesthathavebeeninvestigatedpreviouslyonvariouscancer |
| 194 | cellsandshowedanticanceractivity[29].Inaddition,ourgrouphadinvestigatedcompounds**2**and |
| 195 | **3**againstnon-smallcelllungcancercelllines[10,11].Inthisstudy,compound**2**wasonlyactive |
| 196 | againstPC-3cellline,whilecompound**3**wasonlyinactiveagainstMCF7cellline(**Table1**). With |
| 197 | theexceptionofresultsobtainedforMCF7cellline,compound**3**wasmorecytotoxicthancompound |
| 198 | **2**onalltestedcancercelllines.PC-3celllinewasthemostsensitiveforcompound**3**withIC50value |
| 199 | of3.58µM,followedbyH69cellline(14.20µM),H69ARcellline(35.50µM),andCaco-2cellline |
| 200 | (52.34µM)(**Table1**).AsintheresultsobtainedforDOX,compound**3**wasmoreactiveagainstH69 |
| 201 | celllinecomparedtoH69ARcellline.Ontheotherhand,compound**2**wasinactiveagainstbothcell |
| 202 | lines(H69andH69AR). |
| 203 | Inourpreviouswork [11],wehaveshownthatcompound**3**hasthemostantioxidantactivity |
| 204 | comparedtoLVXandcompound**2**.Wealsohaveshownthatcompounds**2**and**3**inhibitALDH |
| 205 | activitywith compound**3**resultingina significantinhibitioncomparedtocompound**2**[11].Hence, |
| 206 | wehave suggestedthat the possiblemechanismof toxicitymaybe drivenbytheantioxidantactivity |

andALDHinhibition,especiallyforcompound**3**[11].Thiswasfurthersupportedbythemolecular

dockingexperimentsthathaveshownthatcompound**3**hasagoodbindingaffinitytowards

ALDH1A3enzyme[11].

Inthisstudy,intermsofthionatedLVXderivatives,compound**3**showedhighercytotoxic

activitycomparedtocompound**2**onthetestedcancercelllines,especiallyinPC-3cellline.PC-3

celllineisknowntoexpressALDH1A3enzymeandwhenitisknockedout,cells’proliferationhas

beensignificantlyreduced[30].Thiscorrelateswithourresultsinwhichcompound**3**,havinghigh

affinitytowardsALDH1A3[11],resultedinthemostcytotoxiceffectagainstPC-3celllinewithIC50

valueof3.58µM,whileonothercancercelllinesthevaluewashigher(**Table1**).Furthermore,

colorectalcancerresistancetomedicationisdrivenviaALDH1A3upregulation[31]anditsinhibition

mightresultincytotoxicityasitisshownforcompound**3**oncolorectalcancercellline(Caco-2)in

**Table1**.Thismayfurthersupportthemechanism ofcytotoxicityforthesecompoundsoncancercells,

particularlycompound**3**,isdriven, inpart, viaALDHinhibition.

ResearcharticlesusingSCLCcelllinesisveryminimalinliterature.Wecouldnotidentify,

fromresearchpapers,theexpressionofALDHenzymesinSCLCandparticularlyH69orH69AR

celllines.Hence,furtherinvestigationregardingALDHenzymesexpressioninSCLCishighly

neededinthefuture.

TheHumanProteinAtlaswebsite[32],maybeusedtoidentifytheexpressionofspecific

proteinsinseveralcelllinesviaRNAexpressionmeasuredinnormalizedtranscriptpermillion

(nTPM).Amongthetestedcelllinesinthisstudy,PC-3celllineexpressesthehighestlevelof

ALDH1A3withavalueof565.6nTPM,followedbyCaco-2cellline(7.1nTPM),MCF7cellline

(4.8nTPM),andH69cellline(0.7nTPM).However,therewerenodataavailableforH69ARcell

line.Again,thehighexpression ofALDH1A3enzymeinPC-3cellline[32] alongwithcompound **3**

highbindingaffinitytoit[11],furthervalidatesthatthemechanismofcytotoxicityforcompound**3**

isdriven,in part,viaALDHinhibition.

OurfindingssuggestthepossibilityofrepurposingthionatedLVXderivatives,especially

compound**3**,forthetreatmentofcancers,particularlyprostatecancer,duetotheirpromising

cytotoxiceffectsasshownin**Table1**.

3.2.TheUseofDoxorubicininCombinationTreatment

Compound**3**,beingoneofthenovelthionatedLVXderivativeswithhighercytotoxicactivity

comparedtocompound**2**,wasfurtherselectedtoinvestigateitscytotoxicactivityincombination

withDOXonPC-3,MCF7,Caco-2,H69,andH69ARcell lines.

ExperimentswereconductedbytreatingPC-3,MCF7,Caco-2,H69,andH69ARcelllines

for96h.Theconcentrationofcompound**3**wasrangedfrom12.5to0.391µM.Asforcompound**3**

onPC-3cellline,these concentrationswereabove andbelowitsrespectiveIC50valuesonthe tested

celllines(**Table1**).Thisistoinvestigatetheeffectoftoxicandnon-cytotoxicconcentrationsof

compound**3**whencombinedwithDOXonthetestedcelllines.DOX’sconcentrationusedwasator

arounditsrespectiveIC50valueonPC-3cellline(**Table1**).Theresultsarepresentedin**Figure2**.

245

246	**(Figure2here)**

247

248

**Figure2a–e**showsthecytotoxiceffectofcompound**3**incombinationwithDOXcompared

toDOXaloneonPC-3,MCF7,Caco-2,H69,andH69celllines,respectively,inwhichthe

concentrationofDOXwasatorarounditsrespectiveIC50valueforeachcellline(**Table1**).Inall

testedcelllines,therewasanobservedreductionincellviabilitywhencompound**3**wascombined

withDOX,comparedtocompound**3**alone,indicatinganenhancedcytotoxiceffectofthe

combinationtreatments.Moreover,asignificantreductionofcellviability,comparedtoDOXalone

atorarounditsrespective IC50valueoneachcellline,wasobservedonlyinPC-3,H69,and H69AR

celllines.AsignificantdecreaseincellviabilitywasobservedwhenDOXwascombinedwith12.5,

6.25,3.125,and1.563µMofcompound**3**(**Figure2a**);with12.5μMofcompound**3**(**Figure2d**);

andwithallconcentrationsofcompound**3**(**Figure2e**),comparedtoDOXalone.Although

compound**3**washighlycytotoxicagainst PC-3cellline(**Table1**),synergism withDOXwashighly

achievedagainstH69ARcelllineatalltestedconcentrations,whilesynergismwithDOXagainstPC-

3andH69 celllinewasachievedwithrelativelythehigherconcentrationsofcompound**3**.

Interestingly,highsynergismwasachievedwhencompound**3**wascombinedwithDOXon

H69ARcellline,whichisknowntobeDOX-resistantcellline,asmentionedabove.Thiscombination

resultedinhighercytotoxicactivityintheDOX-resistantcelllinecomparedtotheDOX-sensitive

cellline(H69)(**Figure2d**and**2e**).Thismightindicatethepotentialuseofcompound**3**forthe

treatmentofDOX-resistantcancersincombinationtreatmentswithDOX.

Compound**3**mighthaveenhancedDOXcytotoxicactivityorviceversa.Ithasbeen

previouslysuggestedthatALDH-affiniccompoundsmayactasP-glycoproteinsubstrates,thus

allowingmoreDOXentryintothecell,sinceDOXisaP-glycoproteinsubstrateandacompetition

onthebindingsitewould occur[19].However,H69ARcellslackP-glycoproteins[28]thusindicating

thatthissuggestedmechanismofsynergismisinvalid,inpart,forH69ARcelllines.Furthermore,P-

glycoproteinsareexpressedminimallyinPC-3,MCF7,Caco-2,andH69celllinesasobtainedfrom

TheHumanAtlasProteinwebsitewithnTPMvaluesof0.0,0.1,0.0,and0.3,respectively[33].Again,

thepossibilityofcompound**3**toactasP-glycoproteinsubstratewouldnotbevalidtoenhanceDOX’s

cytotoxicityor the reasonforthe enhancedcytotoxicityofthecombination treatment.

ItmightbethatDOXthatisenhancingcompound**3**cytotoxicactivityinamechanismthat

requiresfurtherinvestigation.Ourfindingsfurthershowthepotentialuseandrepurposingof

thionatedLVXderivativesforthetreatmentofcancersaswellastheircombinationwithDOXmay

alsoenhancethecytotoxic effect.

3.3.CalculatingtheCombinationIndexfortheCombinationTreatment

SignificantenhancementofcytotoxicitywasobservedagainstPC-3,H69,andH69ARcell

lines whencompound**3**wascombinedwithDOX(**Figure2**).Theeffectofcombiningcompound**3**

withDOXonPC-3,H69,andH69ARcelllineswasfurtheranalyzedviatheCombinationIndex (CI)

calculations[34].Thecombinationtreatmentsmaybe identifiedas synergistic (CI<1), additive (CI

285	=1),orantagonistic(CI>1)againstthetestedcancercelllines.Theexperimentswouldalsoshow

the changesoftheIC50valuesforeachcompoundwithinthecombinationtreatment.Theresultsare

presentedin**Table2**.

288

**Table2.**IC50values(µM±SEM)forcompound**3**assingleagentandincombinationwithDOX and

forthatofDOXassingleagentandincombinationwithcompound**3**,alongwiththecalculated

combinationindicesforthecombinationtreatmentsonPC-3,H69,andH69ARcelllinesafter

treatmentfor96h;n=3

| **Treatments** | **CellLines** | | |
| --- | --- | --- | --- |
|  | **PC-3** | **H69** | **H69AR** |
| **IC50 for Compound3 as Single**  **Agent** | 3.58 ± 0.16 | 14.20 ± 0.28 | 35.50 ± 0.54 |
| **IC50 for Compound3 in Combination**  **Treatment** | 18.15 ± 0.03 | 3.53 ± 0.13 | 9.82 ± 0.67 |
| **IC50forthe DOX as**  **SingleAgent** | 0.35 ± 0.02 | 0.09 ± 0.01 | 4.62 ± 0.79 |
| **IC50for the DOX in Combination**  **Experiment** | 2.11 ± 0.02 | 0.07 ± 0.00 | 1.48 ± 0.09 |
| **Combination Index** | 11.1* | 1.0** | 0.6*** |

| 293 | SEM:standarderrorofthemean;DOX:doxorubicin;*:antagonisticeffect;**:additiveeffect;***: |
| --- | --- |
| 294 | synergisticeffect |
| 295 |  |
| 296 | AnantagonisticeffectwasobservedagainstPC-3cellline,inwhichaCIvalueof11.1was |
| 297 | achieved.Inaddition,theIC50valuesforcompound**3**andDOXwerehigherthanthoseobtained |
| 298 | whenusedalone.Althoughthismaycontradict theresultsobtainedforPC-3celllineaspresented in |
| 299 | **Figure2**,itmaybethattheconcentrationsusedforcompound**3**withinthisexperimenthaveaffected |
| 300 | thecytotoxiceffectofDOXandthiswouldnotmeaninefficacyin general[11,20]. |
| 301 | Ontheotherhand,theresultsin**Table2**showthattheIC50valuesforcompound**3**andDOX |
| 302 | againstH69andH69AR celllineswerelowercomparedtotheirvalueswhenusedalone,indicating |
| 303 | anenhancementof cytotoxicity.Consequently,theCIvalueagainstH69cellline was1.0indicating |
| 304 | anadditiveeffectofthecombinationtreatmentandaCIvalueof0.6againstH69ARcellline |

indicatingasynergisticeffectofthecombination.Theseresultscanbecorrelatedwiththeresults

obtainedin **Figure2**whichshows asignificantreductionincellviability, especiallyagainstH69AR

cellline.Thisfurthersuggeststhepotentialuseofcompound**3**forthetreatmentofDOX-resistant

cancers.

4. Conclusions

Inconclusion,wehaveshownthatcompound3,athionatedLVXderivative,hasa

concentration-dependentanticanceractivityagainstapanelofcancercelllines,*invitro*,further

indicatingthepossibilityofrepurposingthemforcancertreatment.Ourresultshereandwithinour

previousstudy[11]furthervalidatethatthemechanismofanticanceractivityforthecompound**3**

maybe,inpart,viaALDH1A3inhibition.Inaddition,combiningDOXwithcompound**3**enhanced

thecytotoxicactivityagainstcancercelllines,namelyH69anditsDOX-resultantformH69AR,

formingadditiveandsynergisticeffects,respectively.

317

318	**Abbreviations**

| 319 | ALDH: | aldehydedehydrogenase |
| --- | --- | --- |
| 320 | CI: | combination index |
| 321 | DOX: | doxorubicin |
| 322 | FLZ: | fluconazole |
| 323 | LVX: | levofloxacin |
| 324 | nTPM: | normalizedtranscriptpermillion |
| 325 | SCLC: | smallcelllung cancer |

DataAvailability

Thedatasetsgeneratedduringand/oranalyzedduringthecurrentstudyarepresentedwithinthe

manuscript.

329

330

Acknowledgements

ThisworkwassupportedbyAl-ZaytoonahUniversityofJordangrants(30/06/2024-2025and

27/06/2024-2025).PrincessNourahbintAbdulrahmanUniversityResearchersSupportingProject

number(PNURSP2025R418),PrincessNourahbintAbdulrahmanUniversity,Riyadh,SaudiArabia.

Conflict of Interest

Theauthorsdeclarethattheyhavenoconflictof interestto declare.

Authors’Contributions

Conceptualization,H.A.andO.H.A.;formalanalysis,H.A.andO.H.A.;methodology,H.A.,O.H.A.,

M.A.S.,W.K.,A.I.M.I.,D.H.A.,andS.H.;projectadministration,H.A.,O.H.A.,A.M.B,andM.S.A.;

supervision,H.A.,O.H.A.,A.M.B,andM.S.A.;FundingAcquisition,H.A.,O.H.A.,andA.M.B.;

writing—originaldraft,H.A.andO.H.A.;writing—reviewandediting,H.A.,O.H.A.,M.A.S,and

W.K. Allauthorshavereadandapprovedthemanuscript.Alltheauthorshavereviewedthefinal

versionofthemanuscript andapprovedit for publication.

344

References

1.	EmmersonAM,JonesAM.Thequinolones:decadesofdevelopmentanduse.JAntimicrob

Chemother.2003;51Suppl1:13-20.

2.	MajalekarPP,ShirotePJ.Fluoroquinolones:BlessingsOrCurses.CurrDrugTargets.2020;21(13):1354-

70.

3.	YanK,ZhuM,JiaY,WangJ,CaiY.Efficacyandsafetyofquinolonesvs.otherantimicrobialsforthe

treatmentofuncomplicatedurinarytractinfectionsinadults:asystematicreviewandmeta-analysis.Int

UrogynecolJ.2022;33(5):1103-23.

4.	BartlettJG,DowellSF,MandellLA,FileTM,Jr.,MusherDM,FineMJ.Practiceguidelinesforthe

managementofcommunity-acquiredpneumoniainadults.Infectious DiseasesSocietyofAmerica.ClinInfect

Dis.2000;31(2):347-82.

5.	LandersdorferCB,BulittaJB,KinzigM,HolzgrabeU,SörgelF.Penetrationofantibacterialsintobone:

pharmacokinetic,pharmacodynamicandbioanalyticalconsiderations.ClinPharmacokinet.2009;48(2):89-

124.

6.	KhalilA,WahabA-T,ShafiS,FarooqS,SiddiquiH,ChoudharyM.Studyoftheeffectsofnitroquinoline

derivativesonNeisseriagonorrhoeaecausativeagentofthesexuallytransmittedinfectiongonorrhea.Results

inChemistry.2024;7:101481.

7.	LópezY,TatoM,EspinalP,Garcia-AlonsoF,Gargallo-ViolaD,CantónR,etal.Invitroselectionof

mutantsresistanttoozenoxacincomparedwithlevofloxacinandciprofloxacininGram-positivecocci.J

AntimicrobChemother.2015;70(1):57-61.

8.	HuangCY,YangJL,ChenJJ,TaiSB,YehYH,LiuPF,etal.FluoroquinolonesSuppressTGF-β andPMA-

InducedMMP-9ProductioninCancerCells:ImplicationsinRepurposingQuinoloneAntibioticsforCancer

Treatment.IntJMolSci.2021;22(21).

9.	KhanMA,JabeenR,NastiTH,MohammadO.Enhancedanticryptococcalactivityofchloroquinein

phosphatidylserine-containingliposomesinamurinemodel.JAntimicrobChemother.2005;55(2):223-8.

10.	IbrahimAIM,Abul-FutouhH,BourghliLMS,Abu-SiniM,SunoqrotS,IkhmaisB,etal.Designand

SynthesisofThionatedLevofloxacin:Insights intoa New GenerationofQuinolones withPotentialTherapeutic

andAnalyticalApplications.CurrentIssuesinMolecularBiology.2022;44(10):4626-38.

11.	AbumansourH,AbusaraOH,KhalilW,Abul-FutouhH,IbrahimAIM,HarbMK,etal.Biological

evaluationoflevofloxacinanditsthionatedderivatives:antioxidantactivity,aldehydedehydrogenaseenzyme

inhibition,andcytotoxicityonA549cellline.Naunyn-Schmiedeberg'sArchivesofPharmacology.2024.

12.	Yadav P,ShahK.Quinolines,aperpetual,multipurposescaffoldinmedicinalchemistry.BioorgChem.

2021;109:104639.

13.	NarwantiI,YuZY,SethyB,LaiMJ,LeeHY,OlenaP,etal.6-Regioisomeric5,8-quinolinedionesaspotent

CDC25inhibitorsagainstcolorectalcancers.EurJMedChem.2023;258:115505.

14.	MamidalaA,BokkalaK,ThirukovelaNS,SirassuN,BandariS,NukalaSK.SynthesisofQuinoline-

Morpholine-Coupled	1,2,3-Triazole	Hybrids	as	In	vitro	EGFR	inhibitors.	ChemistrySelect.

2022;7(47):e202203763.

15.	AhadiH,ShokrzadehM,Hosseini-KhahZ,GhassemiBarghiN,GhasemianM,EmamiS.Conversionof

antibacterialquinolonedruglevofloxacintopotentcytotoxicagents.JBiochemMolToxicol.

2023;37(6):e23334.

16.	WuL,LengD, CunD,FogedC,YangM.Advances incombinationtherapyoflungcancer:Rationales,

deliverytechnologiesanddosageregimens.JControlRelease.2017;260:78-91.

17.	FisusiFA,AkalaEO.DrugCombinationsinBreast Cancer Therapy.Pharm Nanotechnol. 2019;7(1):3-

23.

18.	LiuL,HuangX,ShiF,SongJ,GuoC,YangJ,etal.Combinationtherapyforpancreaticcancer:anti-PD-

(L)1-basedstrategy.JExpClinCancerRes.2022;41(1):56.

19.	AbusaraOH,IbrahimAIM,IssaH,HammadAM,IsmailWH.InVitroEvaluationofALDH1A3-Affinic

CompoundsonBreastandProstateCancerCellLinesasSingleTreatmentsandinCombinationwith

Doxorubicin.CurrentIssuesinMolecularBiology.2023;45(3):2170-81.

20.	SunoqrotS,AbusuliehS,AbusaraOH.IdentifyingsynergisticcombinationsofDoxorubicin-Loaded

polyquercetinnanoparticlesandnaturalProducts:Implicationsforbreastcancertherapy.IntJPharm.

2023;645:123392.

21.	Al-AliL,Al-AniRJ,SalehMM,HammadAM,AbuarqoubDA,Abu-IrmailehB,etal.Biologicalevaluation

ofcombinationsoftyrosinekinaseinhibitorswithInecalcitolasnoveltreatmentsforhumanchronicmyeloid

leukemia.SaudiPharmaceuticalJournal.2024;32(2):101931.

22.	HaithamAbusaraO,FreemanS,AojulaHS.Pentapeptidesforthetreatmentofsmallcelllungcancer:

OptimisationbyN(ind)-alkylmodificationofthetryptophansidechain.EurJMedChem.2017;137:221-32.

23.	KciukM,Gielecińska A,MujwarS,KołatD,Kałuzińska-KołatŻ, CelikI,etal.Doxorubicin-AnAgentwith

MultipleMechanismsofAnticancerActivity.Cells.2023;12(4).

24.	CarvalhoC,SantosRX, CardosoS,CorreiaS,OliveiraPJ,SantosMS,etal.Doxorubicin: thegood,the

badandtheuglyeffect.CurrMedChem.2009;16(25):3267-85.

25.	JadidMFS,AghaeiE,TaheriE,SeyyedsaniN,ChavoshiR,AbbasiS,etal.Melatoninincreasesthe

anticancerpotentialofdoxorubicininCaco-2colorectalcancercells.EnvironToxicol.2021;36(6):1061-9.

26.	ATCC. NCI-H69[H69]2024 [cited 2024 6April].Availablefrom:https://www.atcc.org/products/htb-

119#detailed-product-information.

27.	ATCC.H69AR2024[cited20246April].Availablefrom:https://www.atcc.org/products/crl-11351.

28.	MirskiSE,GerlachJH,ColeSP.Multidrugresistanceinahumansmallcelllungcancercelllineselected

inadriamycin.CancerRes.1987;47(10):2594-8.

29.	El-MalahA,YoussefA,IsmailM,KamelM,MahmoudZ.Newpromisinglevofloxacinderivatives:

Design,synthesis,cytotoxicactivityscreening,Topo2βpolymeraseinhibitionassay,cellcycleapoptosisprofile

analysis.BioorgChem.2021;113:105029.

30.	WangS,LiangC,BaoM,LiX,ZhangL,LiS,etal.ALDH1A3correlateswithluminalphenotypein

prostatecancer.TumourBiol.2017;39(4):1010428317703652.

31.	DurinikovaE,KozovskaZ,PoturnajovaM,PlavaJ,CiernaZ,BabelovaA,etal.ALDH1A3upregulation

andspontaneousmetastasisformationisassociatedwithacquiredchemoresistanceincolorectalcancercells.

BMCCancer.2018;18(1):848.

32.	Atlas	THP.	ALDH1A3	2024	[cited	2024	6	April].	Available	from:

https://www.proteinatlas.org/ENSG00000184254-ALDH1A3/cell+line.

33.	ProteinTHA.ATPbindingcassettesubfamilyBmember42024[cited20247April].Availablefrom:

https://www.proteinatlas.org/ENSG00000005471-ABCB4/cell+line.

34.	ChouTC,TalalayP.Quantitativeanalysisofdose-effectrelationships:thecombinedeffectsofmultiple

drugsorenzymeinhibitors.AdvEnzymeRegul.1984;22:27-55. 428

Figure1	Click here to access/download;Figure;Figure 1.tif

Figure2	Click here to access/download;Figure;Figure 2.tif
